# Effect of Black Tea Consumption on Blood Cholesterol: A Meta-Analysis of 15 Randomized Controlled Trials

**DOI:** 10.1371/journal.pone.0107711

**Published:** 2014-09-19

**Authors:** Dongmei Wang, Canhuang Chen, Yu Wang, Jiaxing Liu, Rongkai Lin

**Affiliations:** 1 Department of Laboratory Medicine, No.161 Hospital of PLA, Wuhan City, Hubei Province, PR. China; 2 Department of Cardiology, No. 180 Hospital of PLA, Quanzhou City, Fujian Province, PR. China; 3 Health Management Center, No. 180 Hospital of PLA, Quanzhou City, Fujian Province, PR. China; 4 Department of Urology, No. 180 Hospital of PLA, Quanzhou City, Fujian Province, PR. China; The National Institute for Health Innovation, New Zealand

## Abstract

**Background:**

The results of the studies that have investigated the effects of black tea on blood cholesterol are inconsistent. The aim of this study is to quantitatively assess the effects of black tea on cholesterol concentrations.

**Methods:**

PubMed, Embase, MEDLINE, and Cochrane Library (through to July 2014) were searched for randomized controlled trials (RCTs) designed to investigate the effect of black tea on blood cholesterol concentrations. The study quality was assessed by the Jadad scoring criteria. Pooled effect of black tea consumption on blood cholesterol concentrations was evaluated by fixed-effects or random-effects model. Meta-regression analyses were conducted to estimate dose effects of black tea polyphenols on concentrations of blood cholesterol. Subgroup and sensitivity analyses were performed to assess the potential source of heterogeneity.

**Results:**

The consumption of black tea did not significantly lower TC concentrations either in healthy subjects or patients with coronary artery diseases based on both fixed-effects and random-effects analysis. No significant change was observed in HDL-C concentrations in healthy participants or in subjects with coronary artery disease supplemented with black tea when compared with control participants. The pooled net change of LDL-C in healthy participants was −5.57 mg/dL (95% CI, −9.49 to −1.66 mg/dL; *P* = 0.005) in fixed-effects analysis and −4.56 (95% CI, −10.30 to 1.17 mg/dL; *P* = 0.12) in random-effects analysis. No significant net change was observed in LDL-C concentrations in patients with coronary artery disease. Subgroup and sensitivity did not significantly influence the overall outcomes of this meta-analysis. No significant dose effects of black tea polyphenols on blood cholesterol concentrations were detected in meta-regression analyses.

**Conclusion:**

The meta-analysis suggests that the consumption of black tea might not have beneficial effects on concentrations of TC, HDL-C, and LDL-C. Further high quality RCTs are needed to definitively draw a causal interpretation of the findings.

## Introduction

Currently, cardiovascular diseases (CVD) account for almost 50% of non communicable diseases, which have exceeded communicable diseases as the major disease burden worldwide. CVD is responsible for 17.3 million deaths every year, and remains the primary global death cause [1], [2]. It is projected that 377 billion will be saved by 10% reduction of CVD mortality from 2011 to 2025 [3]. Low high-density lipoprotein-cholesterol (HDL-C) concentrations and high total cholesterol (TC) and low-density lipoprotein-cholesterol (LDL-C) concentrations are the major risk factors of CVD [4]–[8]. A recent study suggests that a 1% reduction of cholesterol can decrease the risk of CVD by 3% [9], whereas hyperlipidemia may increase the heart attack risk by 3-fold [10], indicating that the effective regulation of cholesterol metabolism will reduce the burden of CVD. Accumulating data suggest that healthy foods consumption can significantly lower TC concentration [11], and increase HDL-C concentrations [12]. Therefore, growing attention has been devoted to the dietary intervention on the prevention and treatment of CVD.

Tea, derived from the plant Camellia sinensis, is currently consumed worldwide and considered as a major source of flavonoid consumption in the US diet [13]. Tea is mainly divided into three types, such as green tea, black tea and oolong tea. In general, green tea is produced by non-fermented leaves, while black tea and oolong tea are made from the fermented leaves and partly fermented leaves, respectively [14]. A previous meta-analysis on the basis of 14 randomized controlled trials (RCTs) revealed that green tea consumption can significantly reduce plasma LDL-C and TC concentrations [15]. In addition, the meta-analysis based on observational studies found that tea consumption including green tea, black tea, or oolong tea is significantly associated with the reduction of CVD risk [16]. To date, several RCTs have been designed to evaluate the effects of black tea consumption on blood cholesterol concentrations, with inconsistent results. Although two previous meta-analyses had been conducted to investigate the effect of black tea on blood cholesterol concentrations, one study only included two RCTs when pooling the effects of black tea on TC concentrations [17], while the other study conducted their meta-analysis based on the combined population with different health status [18]. Therefore, we performed this meta-analysis to further assess the effects of black tea on blood cholesterol concentrations based on the PRISMA guidelines.

## Methods

### Literature search

We searched and reviewed the database of PubMed (through to July 2014), Embase (through to July 2014), MEDLINE (through to July 2014), the Cochrane Library (through to July 2014) for RCTs designed to evaluate the effect of black tea on blood cholesterol concentrations in human subjects. All databases were searched using the following terms limited in title or abstract: black tea, black tea extract, catechin, catechins, EGCG, camellia sinensis, theaflavin or tea polyphenols. The results of the searching were restricted to RCTs in human subjects. In addition, we scanned the reference lists of correlated reviews to further identify potential additional articles. The searching process was performed by DM Wang, and CH Chen, and any disagreements were resolved by a third reviewer if needed. A separate search strategy, specific for each electronic database could be found in the Supplemental information (Appendix S1).

### Study selection

Studies were selected in this meta-analysis when meeting the following inclusion criteria: 1) the original study used an appropriate control group for black tea or black tea extract intervention group; 2) the study was a parallel- or crossover-design RCT in humans; 3) subjects ingested black tea or black tea extracts for ≥2 weeks; 4) the study did not use a multi-component treatment containing black tea in the intervention group; 5) the baseline and endpoint values for concentrations of TC, HDL-C, LDL-C or mean differences with either 95% CI, SEM or SD values were shown in the studies.

When necessary, the main authors were contacted for additional information.

### Quality assessment

The quality of the included studies was independently assessed by two authors according to the Jadad scoring criteria: 1) randomization (the trial was described as randomized); 2) reporting the methods of generation of random numbers (using random numbers table, computer, tossed coins or shuffled cards, etc); 3) double-blinding study-design (researcher and participant masking); 4) using a proper allocation concealment; and 5) clearly reporting the number and reasons for dropouts. The included studies achieved one point when meeting the standard of each specific item. The possible score ranged from 0 to 5 (highest quality level) [19]. The high-quality study was with a score of ≥4, whereas the low quality study was with a score of <4.

### Data extraction

The standardized data extraction form of the meta-analysis included the following items: 1) characteristics of each study including the first author, publication year, country of the population, sample size, design of study, duration of study, intervention dose, intervention type and diet type; 2) population information including baseline health status, baseline concentrations of TC, HDL-C, and LDL-C, body mass index (BMI), and mean age of the population; 3) net changes of concentrations of TC, HDL-C, or LDL-C for each group; and 4) all values of cholesterol concentrations were converted to mg/dL (conversion factor: 1 mg/dL = 0.0259 mmol/L) [20]. The data extraction process was independently performed by two authors, and any disagreements were resolved by a third author.

### Statistical analysis

Current meta-analysis was conducted using STATA software (Version 11; StataCorp, College Station, TX). The effective size of intervention was defined as weighted mean difference with its 95% CIs of TC, HDL-C, and LDL-C concentrations. The statistical heterogeneity between individual included trials results was evaluated by Cochran's test and the *I^2^* statistics. The Cochran's test *P* value of <0.10 or the *I^2^* value of >50% suggested a significant heterogeneity between the included studies [21]. A random-effects model (I–V heterogeneity) was used if a significant statistical heterogeneity was detected. Otherwise, a fixed-effects (Inverse Variance) model was applied in the meta-analysis. To provide more credible and detailed results of the data synthesis, we showed the results calculated from both fixed-effects and random-effects models and make the conclusion based on both of the two models.

SD values were indirectly calculated from SEM, *P*-values, 95% CIs, or t-values if they were missing in the original studies. As suggested by Follmann [22], we assumed that the correlation coefficient is 0.5 between the initial and final values.

Subgroup analyses including baseline BMI value, study design, intervention type, intervention duration, black tea polyphenol dose, and study quality were performed to explore the potential source of heterogeneity between the RCTs. Sensitivity analyses were conducted in accordance with the Systematic Review of Interventions Handbook of Cochrane software. Meta-regression analyses were conducted to evaluate the dose-effect of black tea polyphenols on concentrations of TC, HDL-C, or LDL-C. Publication bias in this meta-analysis was evaluated by the funnel plots and Egger's test.

## Results

### Literature Search Results

**Figure 1:**
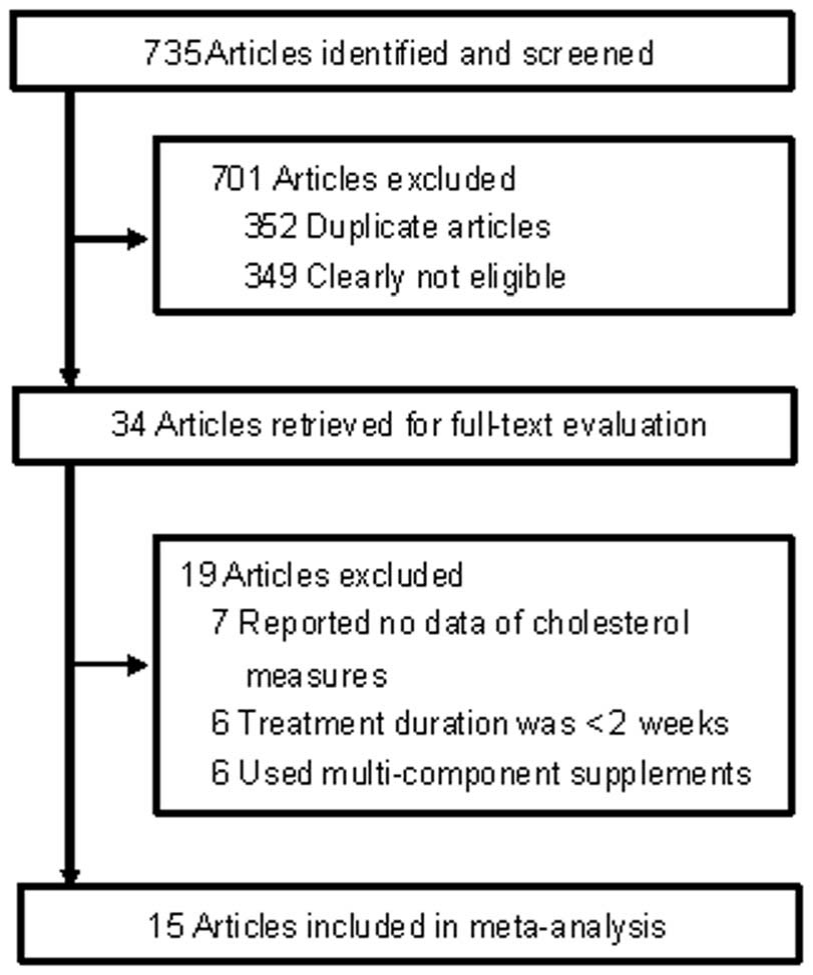
Flow diagram showing the number of studies screened, assessed for eligibility, and included in the meta-analysis.

**Figure 1** showed the detailed processes of selecting the relevant studies. The initial searching yielded a total of 735 potential reports with 701 articles being excluded due to either duplication or being irrelevant to this meta-analysis by carefully reviewing the titles and abstracts of each study. Thus, the remaining 34 articles underwent an in-depth examination. Of these, 7 studies did not report the data of concentrations of TC, HDL-C, or LDL-C, 6 studies had short treatment duration (<2 weeks), and 6 studies used a multi-component supplement containing black tea in intervention group. At last, 15 articles were ultimately included in current meta-analysis [23]–[37].

### Study characteristics

A summary of study characteristics for each included trial was presented in **Table 1**. The sample size of all included trials ranged from 15 to 72. Ten of 15 included studies used parallel design, while the remaining 5 trials used crossover design. Of the 15 trials included in our meta-analysis, 11 selected healthy subjects, 2 included the patients with coronary artery disease, 1 included the subjects with diabetes and 1 included the participants with diabetes or with two other cardiovascular risk factors. Thirteen of the 15 included studies considered cholesterol as a primary outcome, while the remaining 2 considered cholesterol as a secondary outcome [25], [29]. Of the 11 studies including healthy subjects, 5 used black tea extract as supplement, and 6 studies used black tea leaf as intervention supplement. The duration of study varied from 3 weeks to 6 months (median: 4 weeks). In addition, 8 studies provided the information of black tea polyphenols, and the polyphenols content ranged from 77.5 to 1350 mg/d (median: 351.2 mg/d). Most of the trials suggested the subjects to maintain their usual diet, whereas one study provided the same background controlled diet to the volunteers.

**Table 1:** Characteristics of the 14 randomized controlled trials included in the meta-analysis.

Author, publication year, Country	No. of subjects	Study design	Population	Duration	Tea group	Control group	BMI (kg/m^2^)	JadadScore	Type of diet
Bahorun, 2012, Mauritius	72	Parallel	Healthy, 25–60 years of age	12 wk	Black tea leaf (9 g/d)	Water	NR	3	Usual diet
Kubota, 2011, Japan	36	Parallel	Healthy, 52.2±11.9 years of age	12 wk	Black tea extract (1 g/d)	Placebo powder	26.2±2.1	4	Usual diet
Neyestani, 2010, Iran	46	Parallel	T2DM, 57.0±37.9 years of age	4 wk	Black tea extract (2.5 g/d in week 1, increased by 2.5 g/d for each week)	Black tea extract (2.5 g/d)	29.0±1.5	2	Usual diet
Trautwein, 2009, Netherlands	71	Parallel	Healthy, 47.5±6.7 years of age	11 wk	Black tea extract (77.5 mg/d polyphenoles)	Placebo capsules (cellulose)	23.8±2.4	5	Usual diet
Fujita, 2008, Japan	47	Parallel	Healthy with borderline hypercholesterol, 40–70 years of age	3 mo	Black tea extract (1 g/d)	Placebo tablets (dextrin)	23.1±1.5	4	Usual diet
Mukamal, 2007, USA	28	Parallel	Either diabetes or with 2 other cardiovascular risk factors, 66.6±29.9 years of age	6 mo	Black tea extract (318 mg/d polyphenoles)	Water	27.7±16.8	3	Usual diet
Hodgson, 2003, Australia	22	Crossover	Healthy, 59.0±7.5 years of age	4 wk	Black tea leaf (10 g/d)	Water	27.0±2.8	2	Usual diet
Davies, 2003, USA	15	Crossover	Healthy, 53.9±9.3 years of age	3 wk	Black tea leaf (860 mg/d polyphenoles; 203 mg/d caffeine)	Placebo beverage (220 mg/d caffeine)	29.8±5.0	4	Controlled diet
Hodgson, 2002, Australia	20	Parallel	Healthy, 60.9±5.4 years of age	4 wk	Black tea leaf (10 g/d)	Water	28.0±3.2	2	Usual diet
Duffy, 2001a, USA	50	Crossover	Coronary artery disease, 54±8 years of age	4 wk	Black tea leaf (1350 mg/d polyphenoles, 270 mg/d caffeine)	Water	28.4±4.3	3	Usual diet
Duffy, 2001b, USA	49	Crossover	Coronary artery disease, 54.6±9.5 years of age	4 wk	Black tea leaf (1350 mg/d polyphenoles, 270 mg/d caffeine)	Water	30.6±6.3	3	Usual diet

Author, publication year, Country (reference no.)	No. of subjects	Study design	Population	Duration	Tea group	Control group	BMI (kg/m^2^)	Jadad Score	Type of diet
Princen, 1998, Netherlands	31	Parallel	Healthy, 34±12 years of age	4 wk	Black tea extract (326.4 mg/d polyphenoles)	Water	24±3	2	Usual diet
Ishikawa, 1997, Japan	22	Parallel	Healthy, 22±1 years of age	4 wk	Black tea leaf (376 mg/d polyphenoles)	Water	22.8±2.7	2	Usual diet
Bingham, 1997, UK	65	Crossover	Healthy, 40±15 years of age	4 wk	Black tea leaf (six tea bags/d)	Placebo	NR	2	Usual diet
Van het Hof, 1997, Netherland	31	Parallel	Healthy, 20–61 years of age	4 wk	Black tea leaf (215 mg/d polyphenols)	Water	23.8±1.8	2	Usual diet

Abbreviation: BMI, body mass index; NR, not reported; T2DM, type 2 diabetes mellitus.

The usual diet was similar to a conventional diet. The studies by Neyestani (2010), Duffy (2011a), Duffy (2011b) and Bingham (1997) used LDL concentrations calculated based on Friedewald's formula, and the remaining studies used direct measuring method.

### Data quality

Quality of each study was assessed by the Jadad score criteria and the outcome was shown in **Table 1**. Four trials [24], [26], [27], [30] were considered to be of high-quality and 11 studies were deemed as low-quality studies. All of the high-quality RCTs used the adequate allocation concealment and double-blinding in study-design. Of the 4 high-quality studies, one study reported the method of random number generation, and the remaining 3 were only described as randomized. Fourteen of 15 included studies reported the details related to the dropouts unless there was no dropout in the study.

### Effect of black tea on blood cholesterol

**Figure 2:**
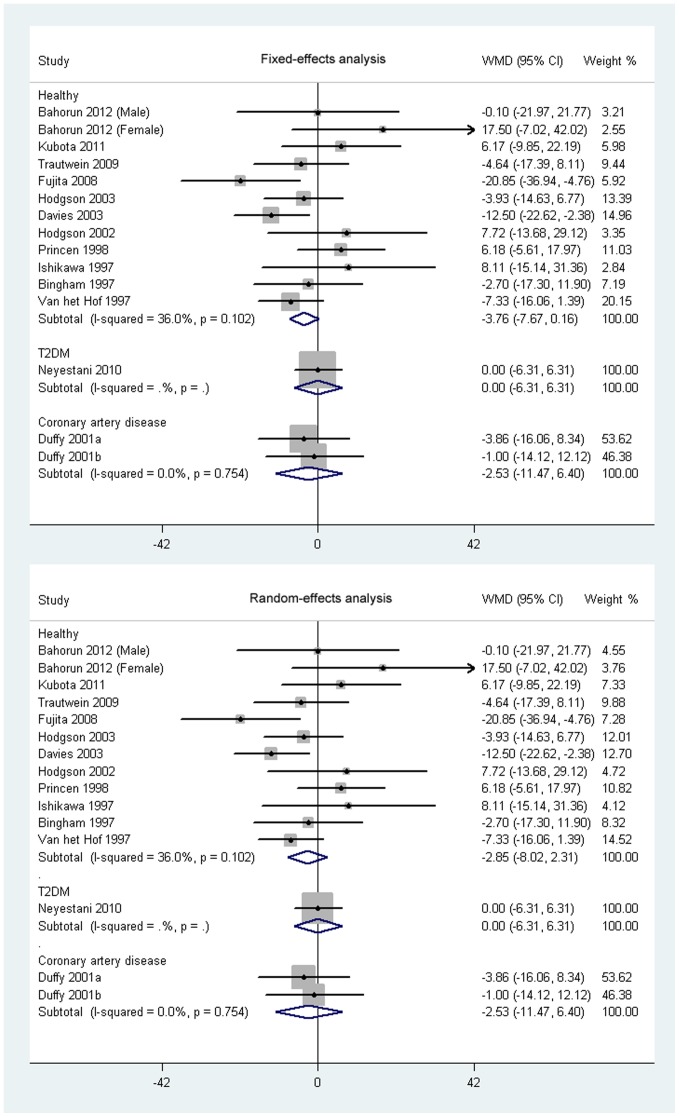
Meta-analysis of effects of black tea on concentrations of total cholesterol (TC). A meta-analysis was done with STATA software (Version 11; StataCorp, College Station, TX). Weight of each study was shown by sizes of data markers in the analysis. The diamond represents the overall estimated outcome and the results were calculated using a fixed-effects or random-effects model. WMD, weighted mean difference.

**Figure 3:**
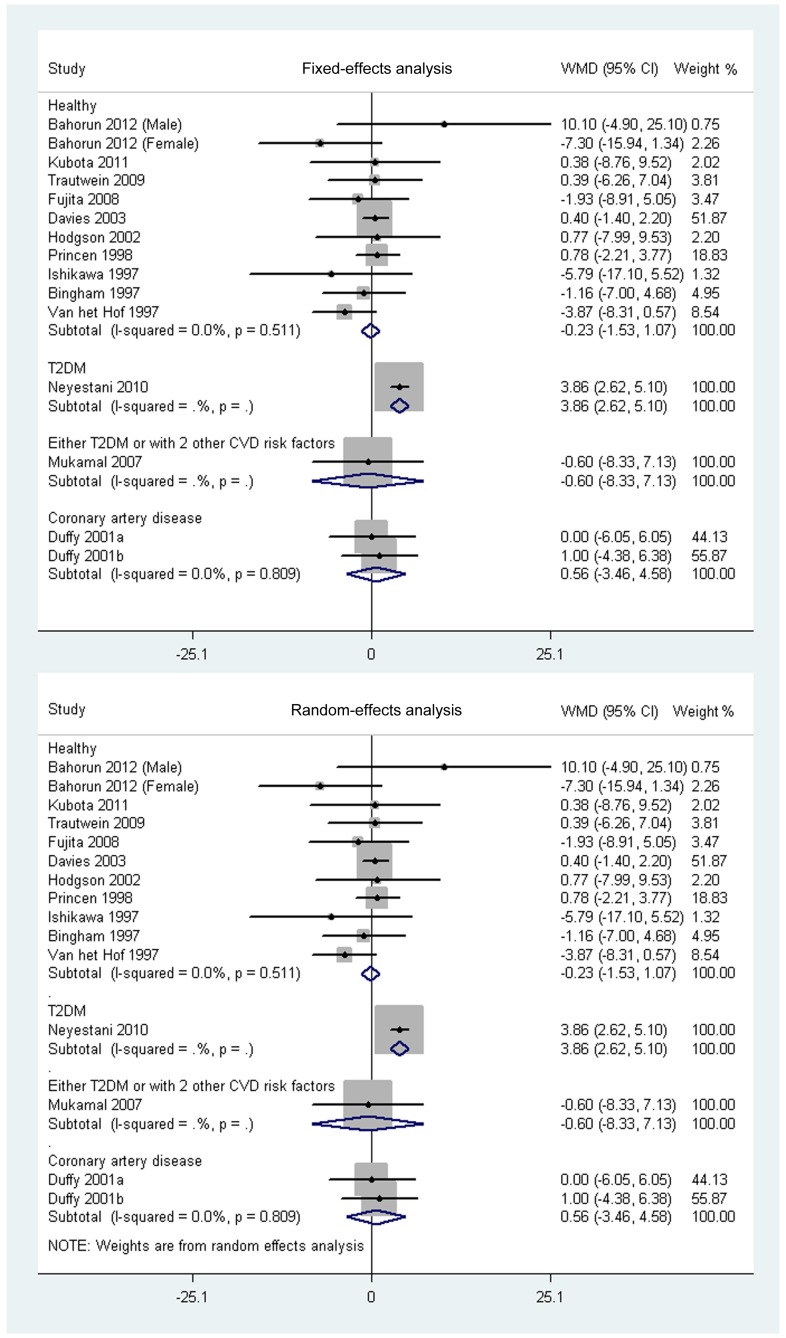
Meta-analysis of effects of black tea on concentrations of high-density lipoprotein-cholesterol (HDL-C). A meta-analysis was done with STATA software (Version 11; StataCorp, College Station, TX). Weight of each study was shown by sizes of data markers in the analysis. The diamond represents the overall estimated outcome and the results were calculated using a fixed-effects or random-effects model. WMD, weighted mean difference.

**Figure 4:**
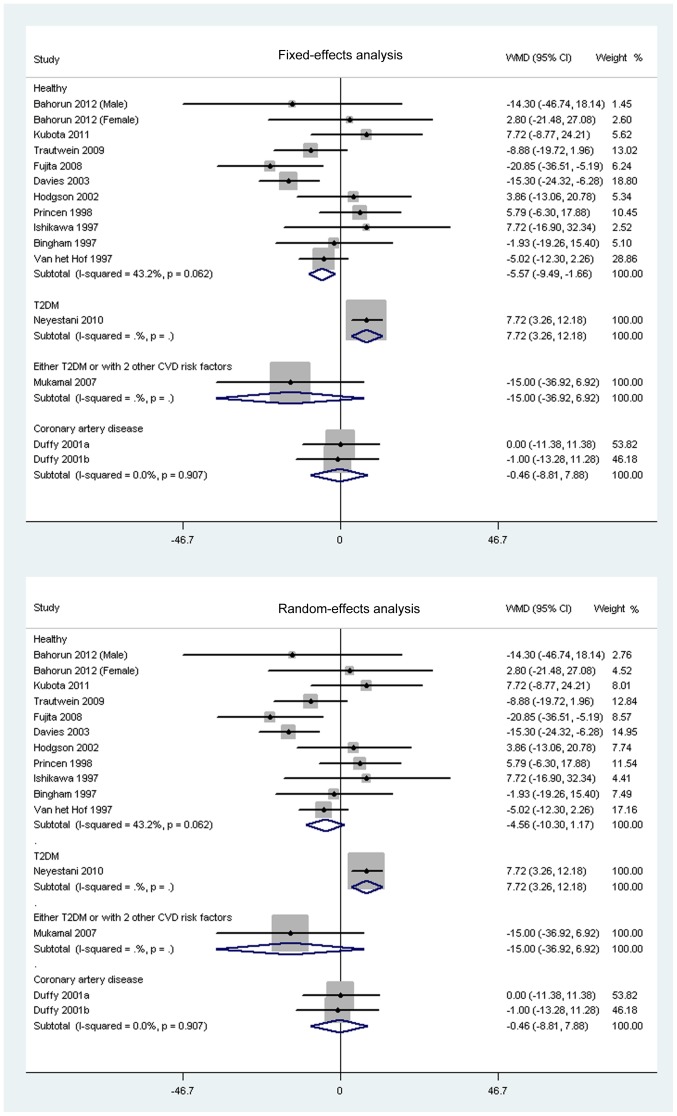
Meta-analysis of effects of black tea on concentrations of low-density lipoprotein-cholesterol (LDL-C). A meta-analysis was done with STATA software (Version 11; StataCorp, College Station, TX). Weight of each study was shown by sizes of data markers in the analysis. The diamond represents the overall estimated outcome and the results were calculated using a fixed-effects or random-effects model. WMD, weighted mean difference.

As shown in **Figure 2**, the consumption of black tea did not significantly lower TC concentrations in healthy subjects based on both fixed-effects analysis (−3.76 mg/dL; 95% CI, −7.67 to 0.16 mg/dL; *P* = 0.06) and random-effects analysis (−2.85 mg/dL; 95% CI, −8.02 to 2.31 mg/dL; *P* = 0.28). Black tea consumption did not significantly affect mean difference change of TC concentrations in patients with coronary artery disease. No significant change was observed in HDL-C concentrations in healthy participants or in subjects with coronary artery disease supplemented with black tea based on both fixed-effects and random-effects analysis when compared with control participants (**Figure 3**). The net change of LDL-C in healthy participants was pooled based on 11 comparisons, and the pooled weighted mean difference was −5.57 mg/dL (95% CI, −9.49 to −1.66 mg/dL; *P* = 0.005; Figure 4) in fixed-effects analysis and −4.56 (95% CI, −10.30 to 1.17 mg/dL; *P* = 0.12; Figure 4) in random-effects analysis. Both the fixed-effects and random-effects analysis were conducted to investigate the effects of black tea on LDL-C concentrations in patients with coronary artery disease, and no significant net change was found (**Figure 4**). In addition, black tea intervention did not show a significant effect on both HDL-C and LDL-C concentrations in participants with diabetes or with two other cardiovascular risk factors (Figure 3 and 4).

Subgroup analyses were conducted to further investigate the influences of baseline BMI, design of study, intervention type, intervention duration, dose of black tea polyphenol, and study quality on the effects of black tea on concentrations of TC, HDL-C and LDL-C in healthy subjects (**Table 2** **–** **4** **and Figure S1–S6**). The intervention duration was categorized as a long-term subgroup (>4 weeks) and a short-term subgroup (≤4 weeks). The treatment dose of black tea polyphenols was classified as either high-dose (>351.2 mg/d) or low-dose (≤351.2 mg/d). As shown in Table 2, subgroup analyses of TC indicated that black tea significantly decreased TC concentrations in crossover design RCTs. However, no significant net change of TC concentrations was detected in other subgroup analyses. Subgroup analyses of HDL-C did not significantly alter the overall effects of black tea on HDL-C concentrations in healthy subjects (Table 3). In the subgroup analyses of LDL-C, the results showed that black tea only significantly lower LDL-C concentrations in the subgroup of studies with high Jadad score based on both fixed-effects and random-effects analysis (Table 4).

**Table 2:** Subgroup analyses of effect of black tea on TC concentrations in healthy subjects stratified by previously defined study characteristics.

Variables	Total cholesterol (mg/dL)				
	No. of comparisons	Net chang (95% CI)	Test of heterogeneity		*P*
			*P*	*I^2^,%*	
**Baseline body mass index**					
** ≥25 kg/m^2^**	5	−3.80 (−9.95, 2.35)^a^	0.17	38.0	0.23
		−2.03 (−10.41, 6.34)^b^			0.63
** <25 kg/m^2^**	4	−5.30 (−11.04, 0.44)^a^	0.06	59.7	0.07
		−5.80 (−15.25, 3.65)^b^			0.23
**Study design**					
** Parallel**	9	−1.81 (−6.69, 3.07)^a^	0.09	41.6	0.47
		−0.58 (−7.46, 6.29)^b^			0.87
** Crossover**	3	−7.29 (−13.86, −0.72)^a^	0.41	0.0	0.03
		−7.29 (−13.86, −0.72)^b^			0.03
**Type of intervention**					
** Black tea leaf**	8	−4.64 (−9.40, 0.13)^a^	0.29	17.6	0.06
		−3.88 (−9.40, 1.64)^b^			0.17
** Black tea extract**	4	−1.92 (−8.81, 4.96)^a^	0.04	63.8	0.58
		−2.80 (−14.44, 8.83)^b^			0.64
**Duration**					
** >4 weeks (high median)**	5	−3.18 (−10.70, 4.35)^a^	0.07	54.6	0.41
		−1.96 (−13.58, 9.65)^b^			0.74
** ≤4 weeks (low median)**	7	−3.97 (−8.56, 0.61)^a^	0.21	28.2	0.09
		−3.28 (−8.93, 2.37)^b^			0.26
**Total polyphenols dose**					
** >351.2 mg/d (high median)**	2	−9.21 (−18.50, 0.07)^a^	0.11	60.6	0.052
		−4.96 (−24.42, 14.50)^b^			0.62
** ≤351.2 mg/d (low median)**	3	−3.04 (−9.18, 3.11)^a^	0.19	40.1	0.33
		−2.51 (−10.66, 5.65)^b^			0.55
**Jadad score**					
** Low (2, 3)**	8	−0.92 (−5.82, 3.99)^a^	0.41	2.9	0.71
		−0.79 (−5.82, 4.24)^b^			0.76
** High (≥4)**	4	−8.74 (−15.24, −2.24)^a^	0.09	53.4	0.01
		−8.24 (−18.09, 1.61)^b^			0.10

a The result was obtained from a fixed-effects model;

b The result was obtained from a random-effects model.

**Table 3:** Subgroup analyses of effect of black tea on HDL-C concentrations in healthy subjects stratified by previously defined study characteristics.

Variables	HDL-C (mg/dL)				
	No. of comparisons	Net change(95% CI)	Test of heterogeneity		*P*
			*P*	*I^2^,%*	
**Baseline body mass index**					
** ≥25 kg/m^2^**	4	0.27 (−1.44, 1.99)^a^	0.77	0.0	0.76
		0.27 (−1.44, 1.99)^b^			0.76
** <25 kg/m^2^**	4	−0.68 (−2.89, 1.53)^a^	0.37	3.7	0.55
		−0.72 (−3.00, 1.56)^b^			0.54
**Study design**					
** Parallel**	9	−0.87 (−2.85, 1.11)^a^	0.41	3.1	0.39
		−0.93 (−2.99, 1.13)^b^			0.38
** Crossover**	2	0.26 (−1.46, 1.99)^a^	0.62	0.0	0.76
		0.26 (−1.46, 1.99)^b^			0.76
**Type of intervention**					
** Black tea leaf**	7	−0.46 (−1.99, 1.07)^a^	0.21	28.8	0.56
		−1.29 (−3.87, 1.29)^b^			0.33
** Black tea extract**	4	0.36 (−2.08, 2.81)^a^	0.92	0.0	0.77
		0.36 (−2.08, 2.81)^b^			0.77
**Duration**					
** >4 weeks (high median)**	5	−1.08 (−4.79, 2.62)^a^	0.35	10.4	0.57
		−1.05 (−5.01, 2.91)^b^			0.60
** ≤4 weeks (low median)**	6	−0.11 (−1.49, 1.28)^a^	0.48	0.0	0.88
		−0.11 (−1.49, 1.28)^b^			0.88
**Total polyphenols dose**					
** >351.2 mg/d (high median)**	2	0.25 (−1.54, 2.03)^a^	0.29	10.9	0.79
		−0.08 (−3.30, 3.15)^b^			0.96
** ≤351.2 mg/d (low median)**	3	−0.54 (−2.87, 1.78)^a^	0.23	32.8	0.65
		−0.78 (−3.85, 2.29)^b^			0.62
**Jadad score**					
** Low (2, 3)**	7	−1.00 (−3.09, 1.08)^a^	0.24	24.5	0.35
		−1.37 (−4.10, 1.36)^b^			0.33
** High (≥4)**	4	0.27 (−1.39, 1.93)^a^	0.94	0.0	0.75
		0.27 (−1.39, 1.93)^b^			0.75

a The result was obtained from a fixed-effects model;

b The result was obtained from a random-effects model.

**Table 4:** Subgroup analyses of effect of black tea on LDL-C concentrations in healthy subjects stratified by previously defined study characteristics.

Variables	LDL-C (mg/dL)				
	No. of comparisons	Net change(95% CI)	Test of heterogeneity		*P*
			*P*	*I^2^,%*	
**Baseline body mass index**					
** ≥25 kg/m^2^**	4	−6.32 (−13.21, 0.56)^a^	0.03	67.5	0.07
		−0.87 (−14.69, 12.96)^b^			0.90
** <25 kg/m^2^**	4	−5.63 (−10.74, −0.52)^a^	0.06	59.6	0.03
		−6.31 (−14.97, 2.34)^b^			0.15
**Study design**					
** Parallel**	9	−3.42 (−7.90, 1.07)^a^	0.15	33.8	0.14
		−2.84 (−8.96, 3.29)^b^			0.36
** Crossover**	2	−12.45 (−20.45, −4.45)^a^	0.18	44.4	0.002
		−10.75 (−23.16, 1.67)^b^			0.09
**Type of intervention**					
** Black tea leaf**	7	−6.43 (−11.29, −1.57)^a^	0.27	20.9	0.01
		−5.71 (−11.85, 0.43)^b^			0.07
** Black tea extract**	4	−4.01 (−10.59, 2.57)^a^	0.02	69.0	0.23
		−4.03 (−16.17, 8.12)^b^			0.52
**Duration**					
** >4 weeks (high median)**	5	−7.46 (−14.73, −0.19)^a^	0.14	42.8	0.04
		−6.89 (−17.39, 3.61)^b^			0.20
** ≤4 weeks (low median)**	6	−4.81 (−9.45, −0.17)^a^	0.07	51.3	0.04
		−3.03 (−10.46, 4.39)^b^			0.42
**Total polyphenols dose**					
** >351.2 mg/d (high median)**	2	−12.58 (−21.05, −4.11)^a^	0.09	66.2	0.004
		−6.76 (−28.55, 15.04)^b^			0.54
** ≤351.2 mg/d (low median)**	3	−3.82 (−9.23, 1.59)^a^	0.19	40.6	0.17
		−3.41 (−10.78, 3.96)^b^			0.37
**Jadad score**					
** Low (2, 3)**	7	−1.20 (−6.41, 4.01)^a^	0.69	0.0	0.65
		−1.20 (−6.41, 4.01)^b^			0.65
** High (≥4)**	4	−11.22 (−17.13, −5.30)^a^	0.06	59.9	0.001
		−10.13 (−20.02, −0.25)^b^			0.04

a The result was obtained from a fixed-effects model;

b The result was obtained from a random-effects model.

Meta-regression analyses indicated a non-significant dose-responsive effects of black tea polyphenols on TC concentrations (*P* for trend  = 0.61), HDL-C concentrations (*P* for trend  = 0.92) or LDL-C concentrations (*P* for trend  = 0.83). The sensitivity analyses showed that the overall outcome of black tea on blood cholesterol concentrations were not significantly affected after imputation a correlation coefficient of 0.5. Moreover, systematically removing each trial during the sensitivity analyses did not significantly affect the overall outcomes of black tea on the concentration of TC, HDL-C or LDL-C (**Figure S7–S9**).

### Publication bias

The funnel plots were symmetrical and the Egger's tests showed no significant publication bias in our meta-analyses of TC, HDL-C and LDL-C concentrations (Egger's test: *P* = 0.09, 0.38 and 0.37, respectively).

## Discussion

The overall outcome of this meta-analysis suggested that black tea intake did not significantly alter concentrations of TC, HDL-C, or LDL-C in healthy subjects. The results of subgroup analyses of TC, HDL-C, and LDL-C did not significantly affect the overall outcome of the effect of black tea on these biomarkers. Although subgroup analyses of LDL-C in healthy subjects indicated that administration of black tea significantly lower LDL-C concentrations based on the studies with high Jadad socre, significant heterogeneity was found in this subgroup. Due to the limited number of included studies, we could not conduct subgroup analysis to confirm the effects of black tea on blood cholesterol concentrations in subjects with T2DM and coronary artery diseases and thus these results should be confirmed by more RCTs in the future.

A previous meta-analysis pooling the data of observational studies suggested that 1 cup/d of green tea rather than black tea is correlated to a 10% reduction in the risk of coronary artery diseases development [38]. In addition, a large cohort study also found that green tea consumption, rather than black tea, is significantly associated with a decreased risk of mortality from CVD [39]. Recently, Zhao et al. conducted a meta-analysis investigating the effect of black tea on blood cholesterol concentrations on the basis of 10 RCTs. However, the overall meta-analysis and subgroup analyses of the study were based on the combined populations with different health status, which might limit the drawing of conclusions about the specific populations. In general, this meta-analysis indicated that black tea consumption has no significant effect on blood cholesterol concentrations, while a previous meta-analysis suggested that green tea consumption can significantly lower the blood TC and LDL-C concentrations [15]. Therefore, black tea and green tea consumption might possess inconsistent effect on cholesterol concentrations and CVD risk. The null effect of black tea on blood cholesterol may be partly because black tea contains less antioxidant compounds than green tea [40]. Additionally, the amount and composition of catechins are substantially various between black tea and green tea due to the different degrees of fermentation. It has been reported that catechins constitute 80% to 90% of total green tea flavonoids, whereas they only constitute 20% to 30% of black tea flavonoids [41]–[43]. This is mainly due to the fact that black tea catechins are usually converted to some complex varieties, such as thearubigins and theaflavins during the oxidation process in the manufacture of black tea [14], [43]. Animal studies have revealed that administration of catechins can significantly increase the activity of hepatic LDL-receptor and reduce plasma and liver cholesterol concentrations [9], [10]. In addition, Chan et al. suggested that catechins can inhibit cholesterol absorption by enhancing the cholesterol fecal excretion in hamsters [44]. Consistent with the animal studies, a previous *in vitro* study has suggested that catechins can directly inhibit the biosynthesis of cholesterol by selectively inhibiting the activity of squalene epoxidase [45]. Thus, if catechins account for the major beneficial effect of green tea on cholesterol concentrations [15], the comparatively lacking favorable effect of black tea on blood cholesterol concentrations is reasonable. In addition, all of the included studies selected participants with TC concentrations lower than 240 mg/dL, and most of the trials (14 of 15) included subjects with high concentrations of HDL-C (higher than 40 mg/dL). This may also partially explain the null effects of black tea on TC and HDL-C concentrations, because TC and HDL-C concentrations may fluctuate in a certain range in the subjects with normal cholesterol conditions [46]. Therefore, black tea consumption may not significantly affect the physiological regulation of blood cholesterol concentrations in these subjects. It has been suggested that the indirect calculation of LDL concentrations based on the Friedewald's formula, might generate uninterpretable calculated values in the participants with highly abnormal triglycerides [47]. In this meta-analysis, 4 studies [25], [32], [33], [36] of the 15 included studies used Friedewald's formula to calculate LDL-C concentrations, and the remaining 11 studies used direct measuring method to detect LDL-C concentrations. However, the mean triglycerides concentrations of the subjects in the four studies ranged from 93 to 275 mg/dL, which may not be considered as highly abnormal [48]. It has been demonstrated that the results of direct measuring method (x) of LDL-C and the Friedewald's formula (y) were highly correlated (r = 0.9908, y = 1.030x−0.289) when the TC concentrations ranged from 60 to 308 mg/dL [48]. Therefore, we did not exclude the studies using Friedewald's formula to calculate LDL-C concentrations and perform further analysis to investigate the influence of triglycerides concentrations on the meta-analysis on LDL-C concentrations.

Although we believe that this study provides useful findings, several inevitable limitations should be addressed. First, of the 15 studies, only 4 were identified as high-quality RCTs by Jadad scoring criteria, whereas the remaining 11 were of low-quality. This is mainly due to that 9 of 15 included studies used water as placebo in the control group, which is difficult for the researchers to conduct double-blinding.

Second, only one study provided the same background controlled diet to both intervention and control group during the study period. Most of the studies only suggested the participants keep their usual diet and limit consumption of black tea, caffeine, or polyphenols, etc. Due to the wide range and distribution of polyphenols in foods and drinks, the precise control of dietary intake in the original studies including free-living subjects was impossible. The differences in background dietary intake might bring confounding factors that affect the current results of this meta-analysis.

Third, we cannot independently conduct meta-analyses to explore the effect of black tea polyphenols on blood cholesterol concentrations because caffeine is naturally existed in black tea and there is limited information about the content of caffeine in most of the included studies. Therefore, it is hard for us to evaluate the potential confounding effect of caffeine on cholesterol concentrations.

In addition, it is difficult for us to evaluate the interaction between black tea consumption and medicine use in subjects with diabetes or coronary artery diseases. However, none of the included studies reported significant unsafe effects of black tea on included participants. Moreover, measures of cholesterol were not the primary outcome in part of the RCTs reviewed in the meta-analysis and the null findings of secondary outcomes may not always be published.

In conclusion, this meta-analysis showed that black tea consumption may have no significant effect on TC, HDL-C, and LDL-C concentrations. Further high quality RCTs are needed to definitively draw a causal interpretation of the findings.

## Supporting Information

Figure S1 **Subgroup analyses of effect of black tea on TC concentrations in healthy subjects stratified by previously defined study characteristics based on the fixed-effects analysis.** A meta-analysis was done with STATA software (Version 11; StataCorp, College Station, TX). Weight of each study was shown by sizes of data markers in the analysis. The diamond represents the overall estimated outcome. NR, not report; WMD, weighted mean difference.
(TIF)

Figure S2 **Subgroup analyses of effect of black tea on TC concentrations in healthy subjects stratified by previously defined study characteristics based on the random-effects analysis.** A meta-analysis was done with STATA software (Version 11; StataCorp, College Station, TX). Weight of each study was shown by sizes of data markers in the analysis. The diamond represents the overall estimated outcome. NR, not report; WMD, weighted mean difference.
(TIF)

Figure S3 **Subgroup analyses of effect of black tea on HDL-C concentrations in healthy subjects stratified by previously defined study characteristics based on the fixed-effects analysis.** A meta-analysis was done with STATA software (Version 11; StataCorp, College Station, TX). Weight of each study was shown by sizes of data markers in the analysis. The diamond represents the overall estimated outcome. NR, not report; WMD, weighted mean difference.
(TIF)

Figure S4 **Subgroup analyses of effect of black tea on HDL-C concentrations in healthy subjects stratified by previously defined study characteristics based on the random-effects analysis.** A meta-analysis was done with STATA software (Version 11; StataCorp, College Station, TX). Weight of each study was shown by sizes of data markers in the analysis. The diamond represents the overall estimated outcome. NR, not report; WMD, weighted mean difference.
(TIF)

Figure S5 **Subgroup analyses of effect of black tea on LDL-C concentrations in healthy subjects stratified by previously defined study characteristics based on the fixed-effects analysis.** A meta-analysis was done with STATA software (Version 11; StataCorp, College Station, TX). Weight of each study was shown by sizes of data markers in the analysis. The diamond represents the overall estimated outcome. NR, not report; WMD, weighted mean difference.
(TIF)

Figure S6 **Subgroup analyses of effect of black tea on LDL-C concentrations in healthy subjects stratified by previously defined study characteristics based on the random-effects analysis.** A meta-analysis was done with STATA software (Version 11; StataCorp, College Station, TX). Weight of each study was shown by sizes of data markers in the analysis. The diamond represents the overall estimated outcome. NR, not report; WMD, weighted mean difference.
(TIF)

Figure S7 **Sensitivity analyses of effect of black tea on TC concentrations in healthy subjects.** A meta-analysis was done with STATA software (Version 11; StataCorp, College Station, TX). Weight of each study was shown by sizes of data markers in the analysis. The diamond represents the overall estimated outcome and the results were calculated using a fixed-effects or random-effects model. WMD, weighted mean difference.
(TIF)

Figure S8 **Sensitivity analyses of effect of black tea on HDL-C concentrations in healthy subjects.** A meta-analysis was done with STATA software (Version 11; StataCorp, College Station, TX). Weight of each study was shown by sizes of data markers in the analysis. The diamond represents the overall estimated outcome and the results were calculated using a fixed-effects or random-effects model. WMD, weighted mean difference.
(TIF)

Figure S9 **Sensitivity analyses of effect of black tea on LDL-C concentrations in healthy subjects.** A meta-analysis was done with STATA software (Version 11; StataCorp, College Station, TX). Weight of each study was shown by sizes of data markers in the analysis. The diamond represents the overall estimated outcome and the results were calculated using a fixed-effects or random-effects model. WMD, weighted mean difference.
(TIF)

Appendix S1 **A separate search strategy designed for each electronic database.**
(DOC)

Appendix S2 **Supporting PRISMA Flow Diagram.**
(PDF)

Checklist S1 **Supporting PRISMA checklist.**
(PDF)
