# Supplementary figures and images for: Effect of Black Tea Consumption on Blood Cholesterol: A Meta-Analysis of 15 Randomized Controlled Trials
Source: PLoS One. 2014 Sep 19;9(9):e107711. doi: 10.1371/journal.pone.0107711 (PMC4169558; doi:10.1371/journal.pone.0107711)

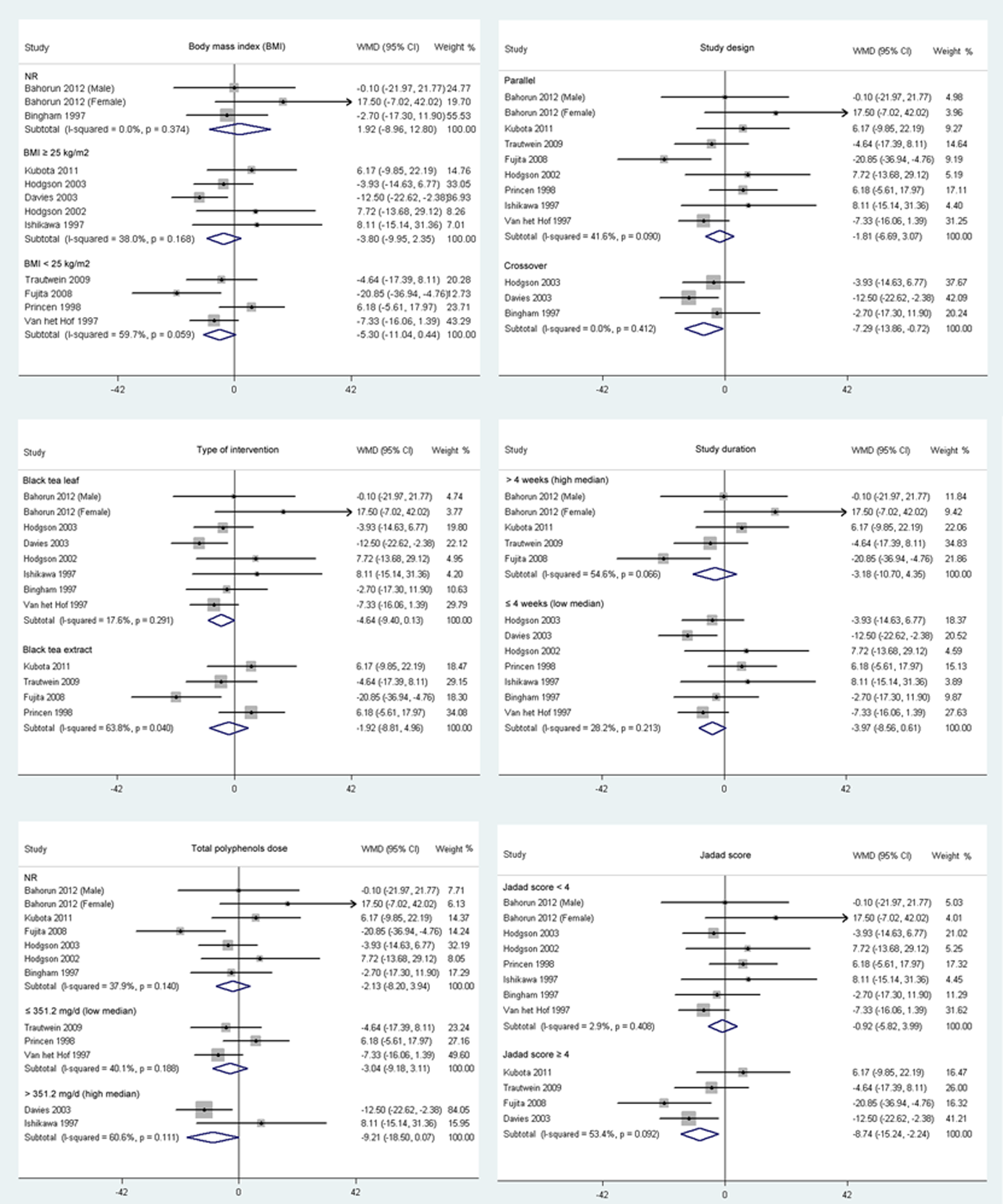

Supplement: Figure S1 — Subgroup analyses of effect of black tea on TC concentrations in healthy subjects stratified by previously defined study characteristics based on the fixed-effects analysis. A meta-analysis was done with STATA software (Version 11; StataCorp, College Station, TX). Weight of each study was shown by sizes of data markers in the analysis. The diamond represents the overall estimated outcome. NR, not report; WMD, weighted mean difference. (TIF) [file pone.0107711.s001.tif]

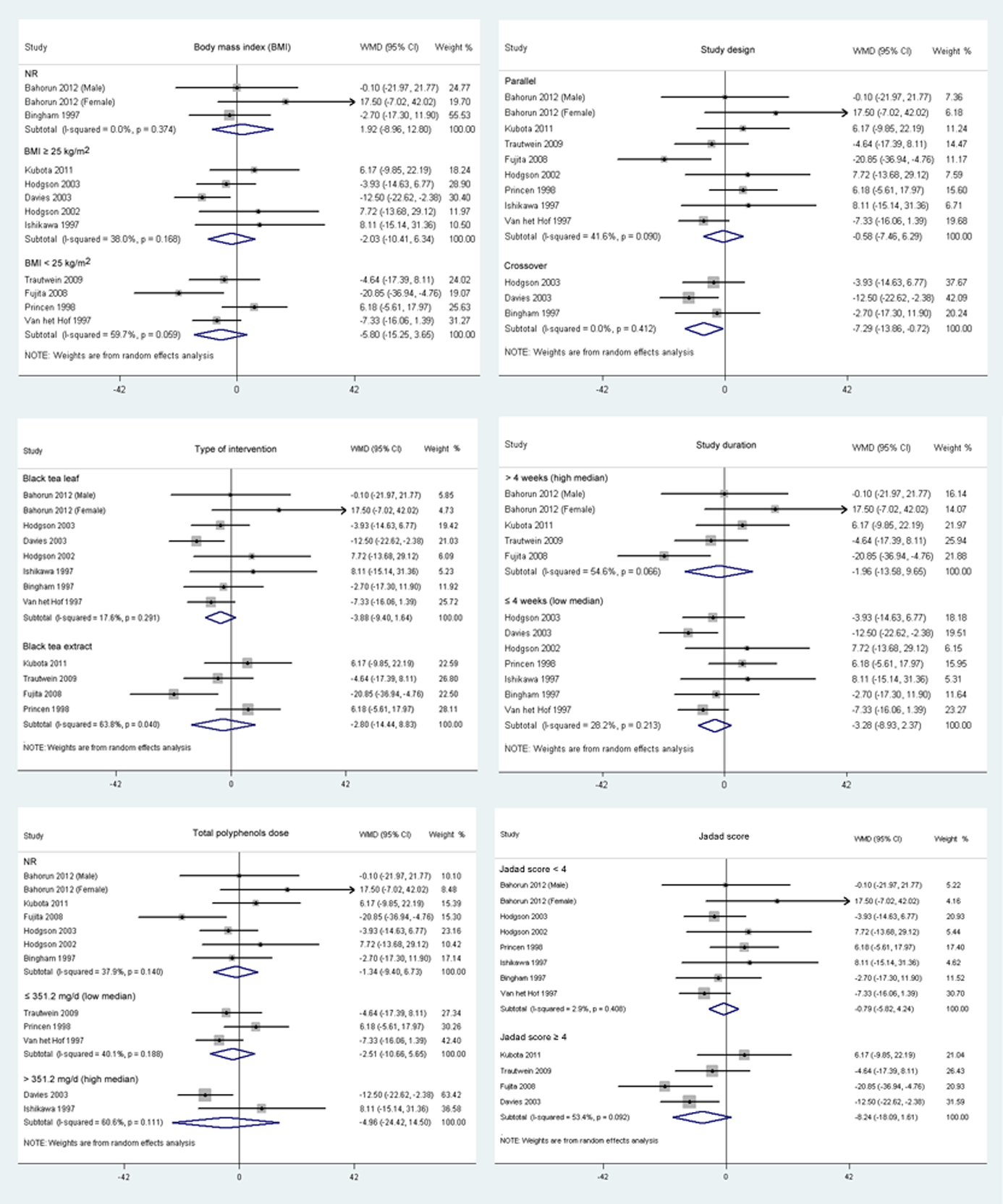

Supplement: Figure S2 — Subgroup analyses of effect of black tea on TC concentrations in healthy subjects stratified by previously defined study characteristics based on the random-effects analysis. A meta-analysis was done with STATA software (Version 11; StataCorp, College Station, TX). Weight of each study was shown by sizes of data markers in the analysis. The diamond represents the overall estimated outcome. NR, not report; WMD, weighted mean difference. (TIF) [file pone.0107711.s002.tif]

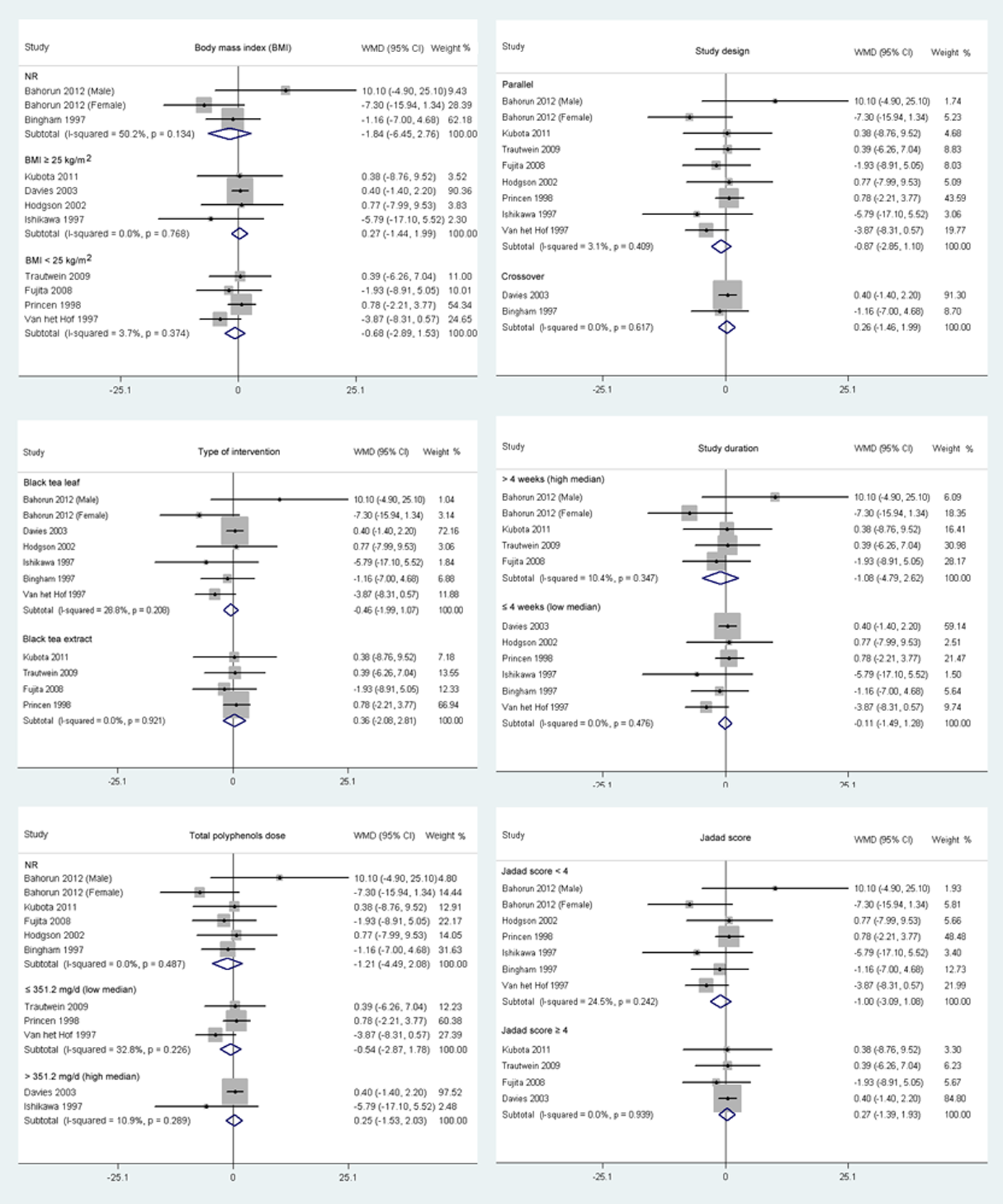

Supplement: Figure S3 — Subgroup analyses of effect of black tea on HDL-C concentrations in healthy subjects stratified by previously defined study characteristics based on the fixed-effects analysis. A meta-analysis was done with STATA software (Version 11; StataCorp, College Station, TX). Weight of each study was shown by sizes of data markers in the analysis. The diamond represents the overall estimated outcome. NR, not report; WMD, weighted mean difference. (TIF) [file pone.0107711.s003.tif]

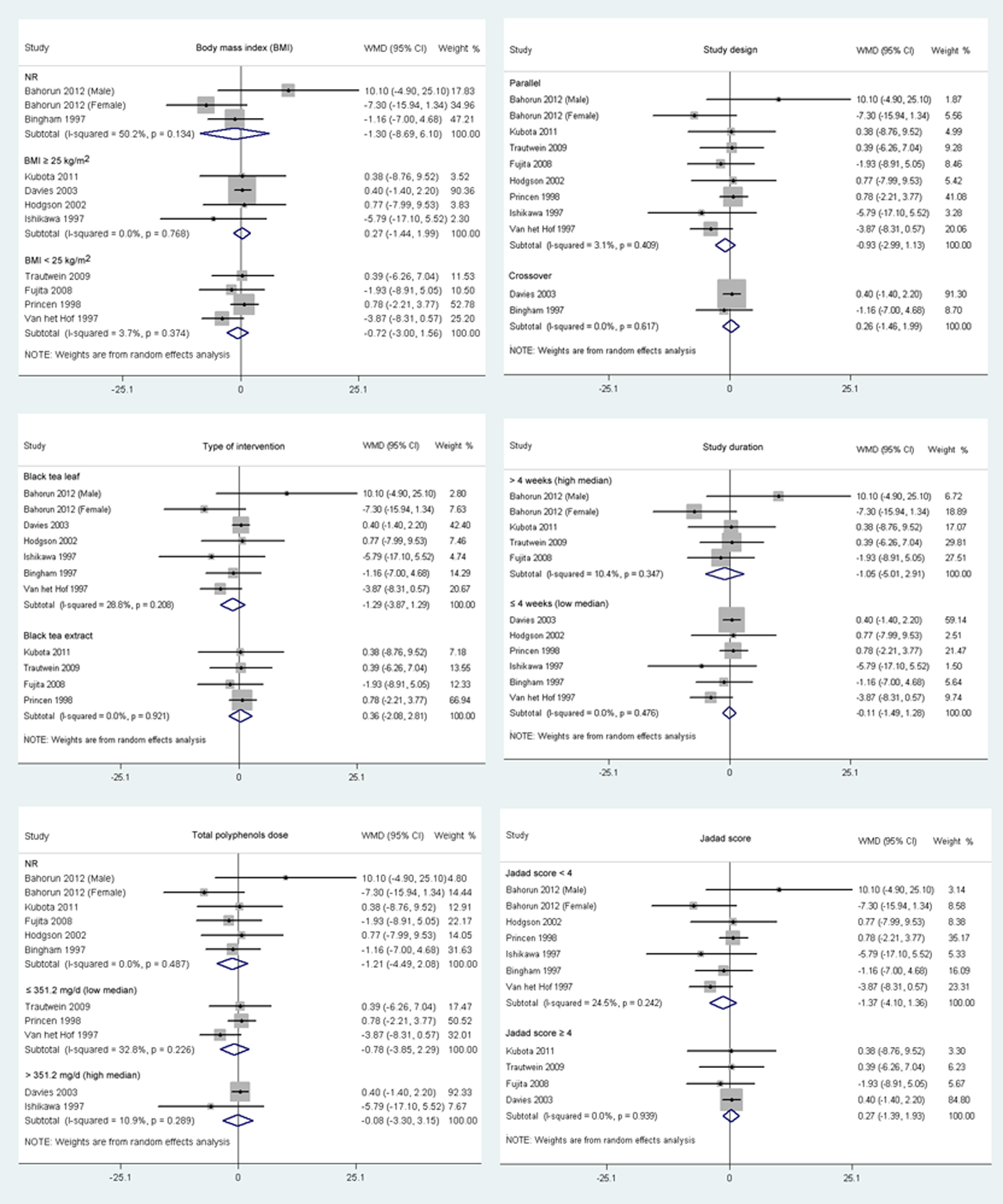

Supplement: Figure S4 — Subgroup analyses of effect of black tea on HDL-C concentrations in healthy subjects stratified by previously defined study characteristics based on the random-effects analysis. A meta-analysis was done with STATA software (Version 11; StataCorp, College Station, TX). Weight of each study was shown by sizes of data markers in the analysis. The diamond represents the overall estimated outcome. NR, not report; WMD, weighted mean difference. (TIF) [file pone.0107711.s004.tif]

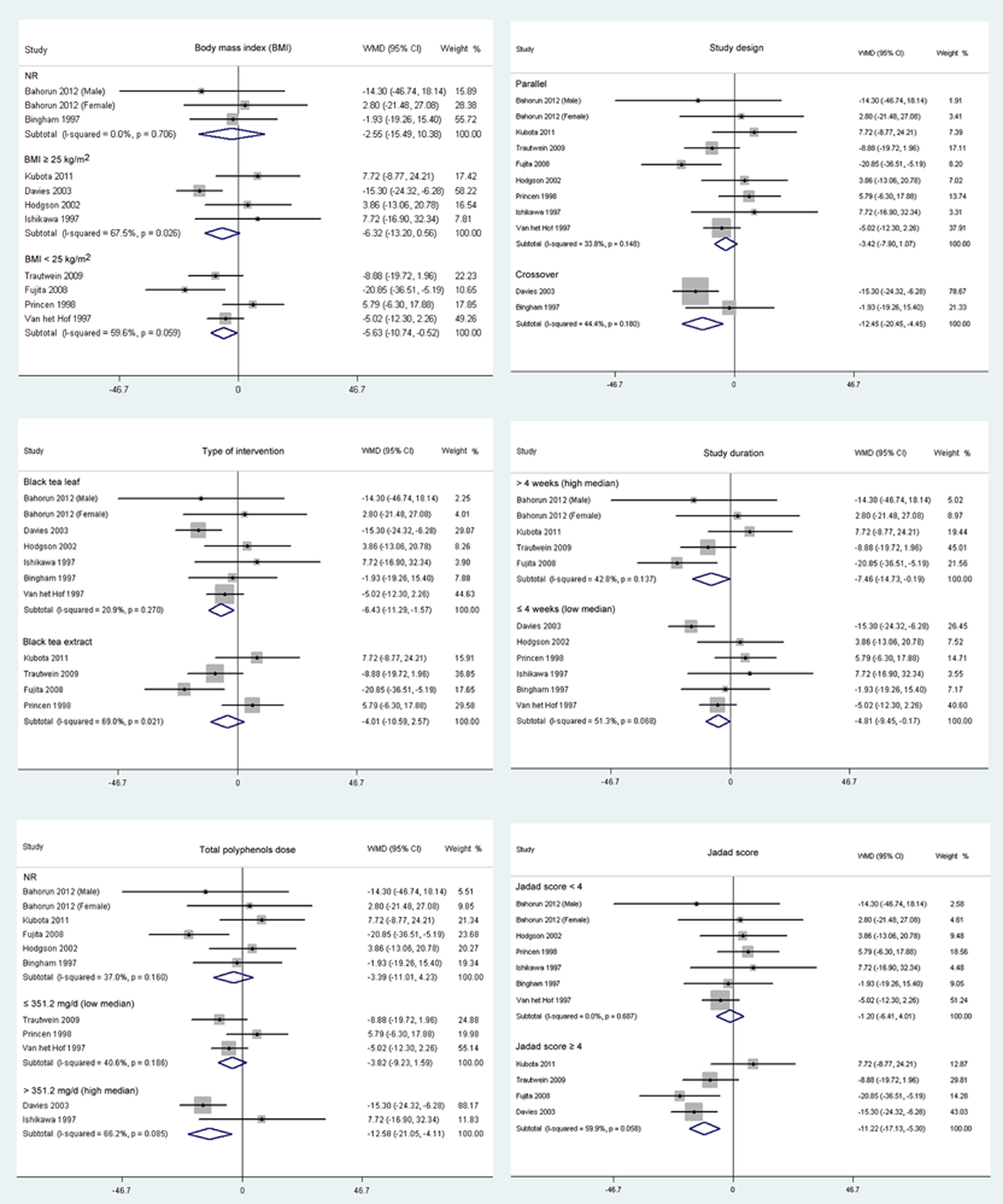

Supplement: Figure S5 — Subgroup analyses of effect of black tea on LDL-C concentrations in healthy subjects stratified by previously defined study characteristics based on the fixed-effects analysis. A meta-analysis was done with STATA software (Version 11; StataCorp, College Station, TX). Weight of each study was shown by sizes of data markers in the analysis. The diamond represents the overall estimated outcome. NR, not report; WMD, weighted mean difference. (TIF) [file pone.0107711.s005.tif]

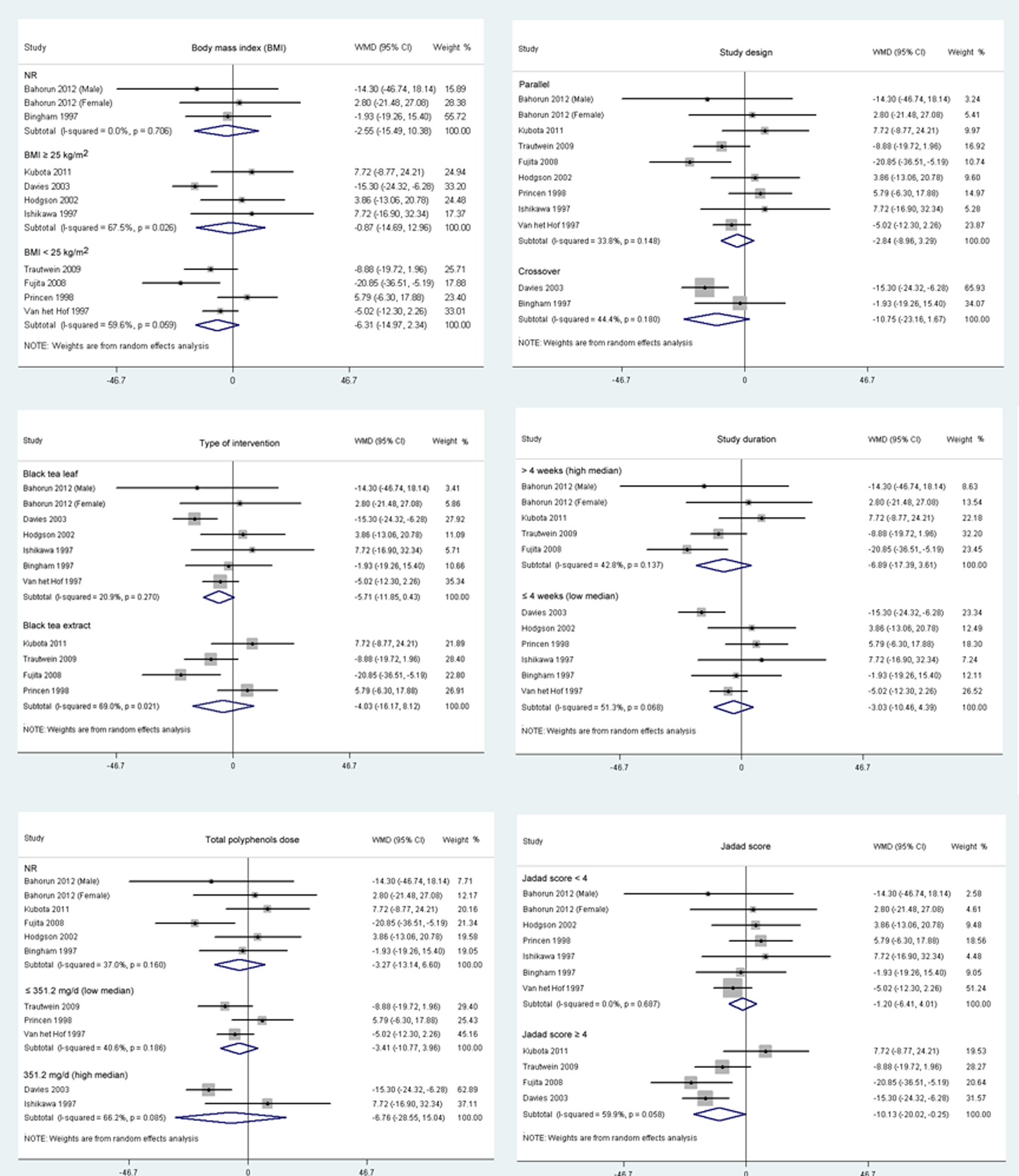

Supplement: Figure S6 — Subgroup analyses of effect of black tea on LDL-C concentrations in healthy subjects stratified by previously defined study characteristics based on the random-effects analysis. A meta-analysis was done with STATA software (Version 11; StataCorp, College Station, TX). Weight of each study was shown by sizes of data markers in the analysis. The diamond represents the overall estimated outcome. NR, not report; WMD, weighted mean difference. (TIF) [file pone.0107711.s006.tif]

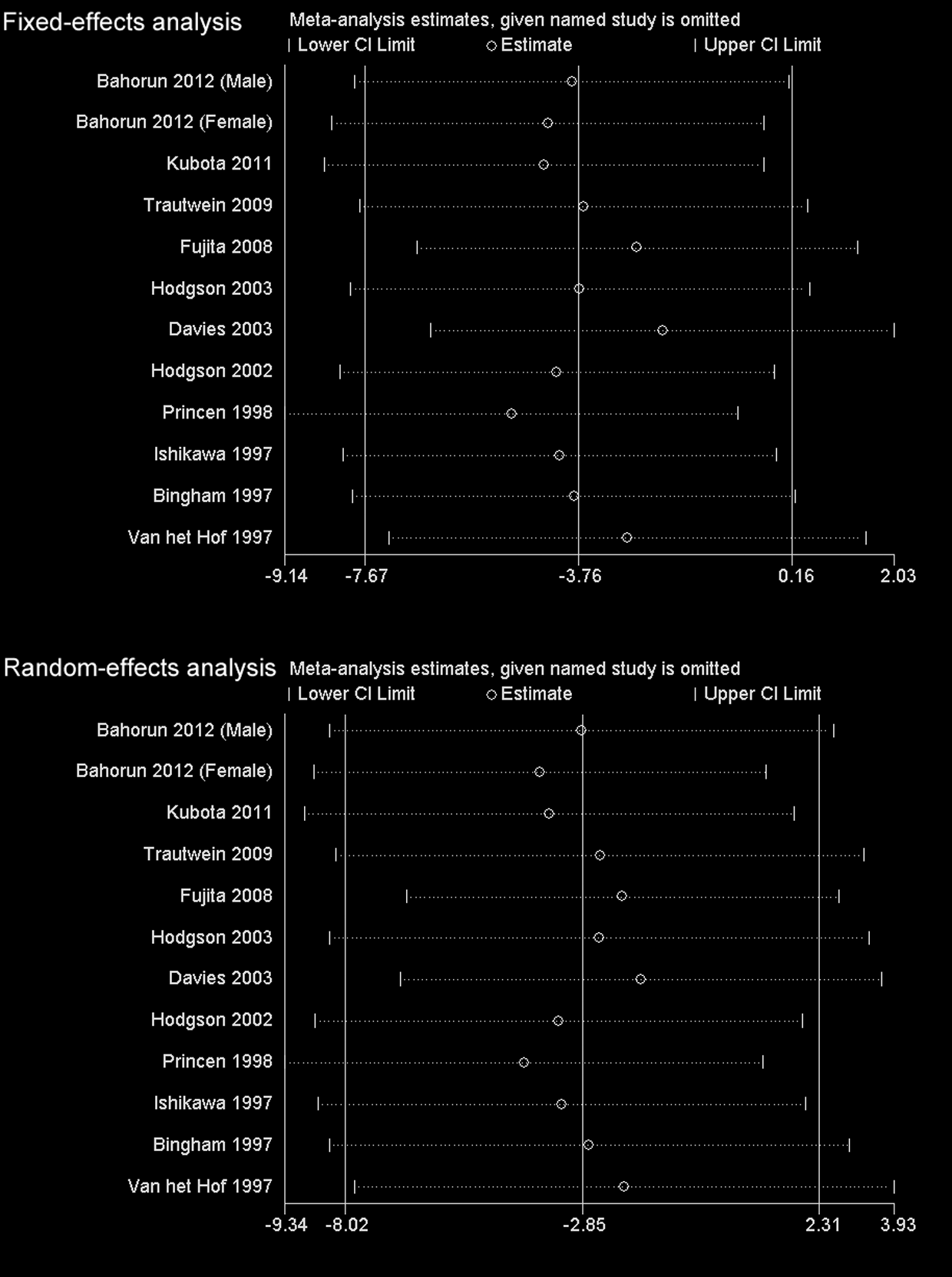

Supplement: Figure S7 — Sensitivity analyses of effect of black tea on TC concentrations in healthy subjects. A meta-analysis was done with STATA software (Version 11; StataCorp, College Station, TX). Weight of each study was shown by sizes of data markers in the analysis. The diamond represents the overall estimated outcome and the results were calculated using a fixed-effects or random-effects model. WMD, weighted mean difference. (TIF) [file pone.0107711.s007.tif]

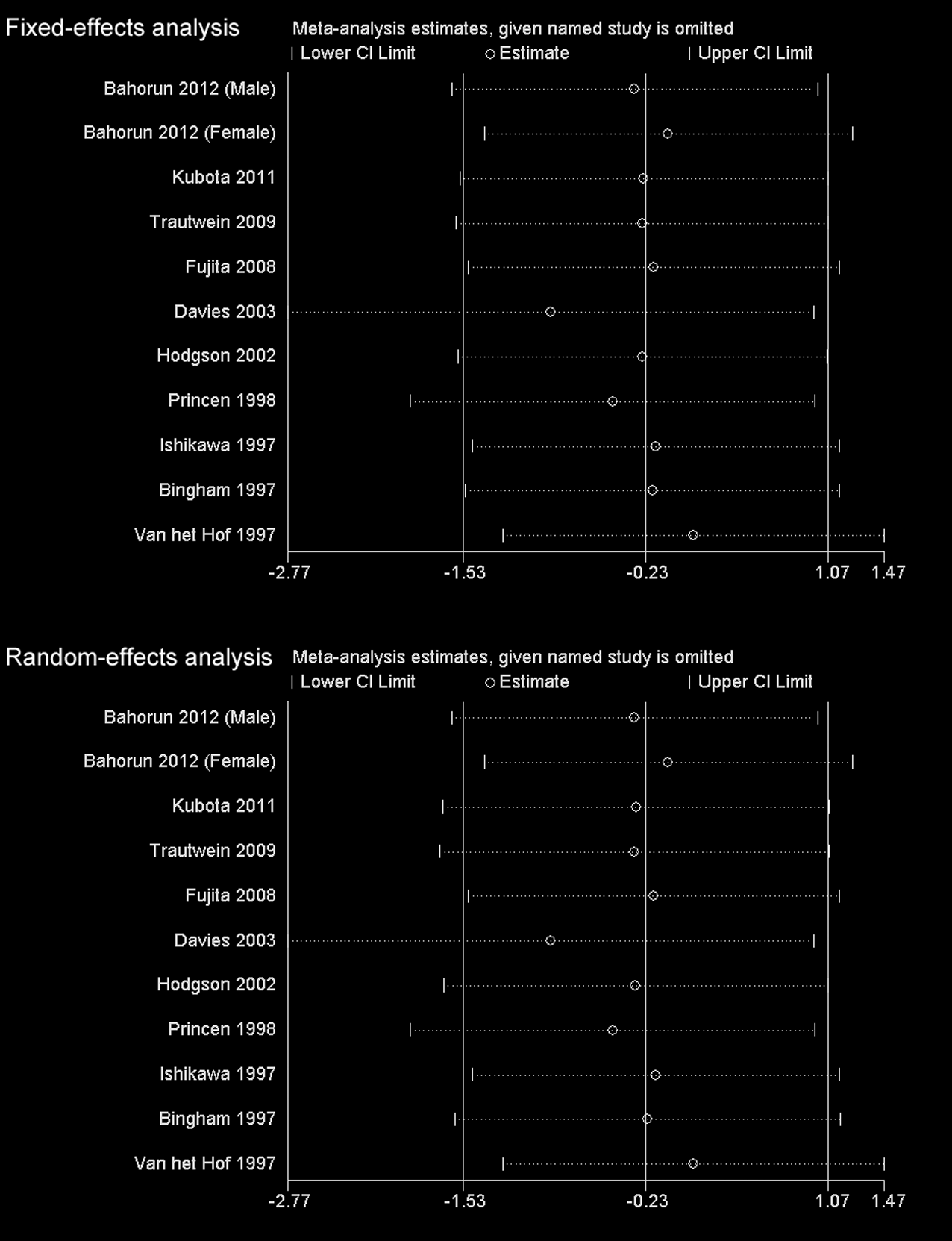

Supplement: Figure S8 — Sensitivity analyses of effect of black tea on HDL-C concentrations in healthy subjects. A meta-analysis was done with STATA software (Version 11; StataCorp, College Station, TX). Weight of each study was shown by sizes of data markers in the analysis. The diamond represents the overall estimated outcome and the results were calculated using a fixed-effects or random-effects model. WMD, weighted mean difference. (TIF) [file pone.0107711.s008.tif]

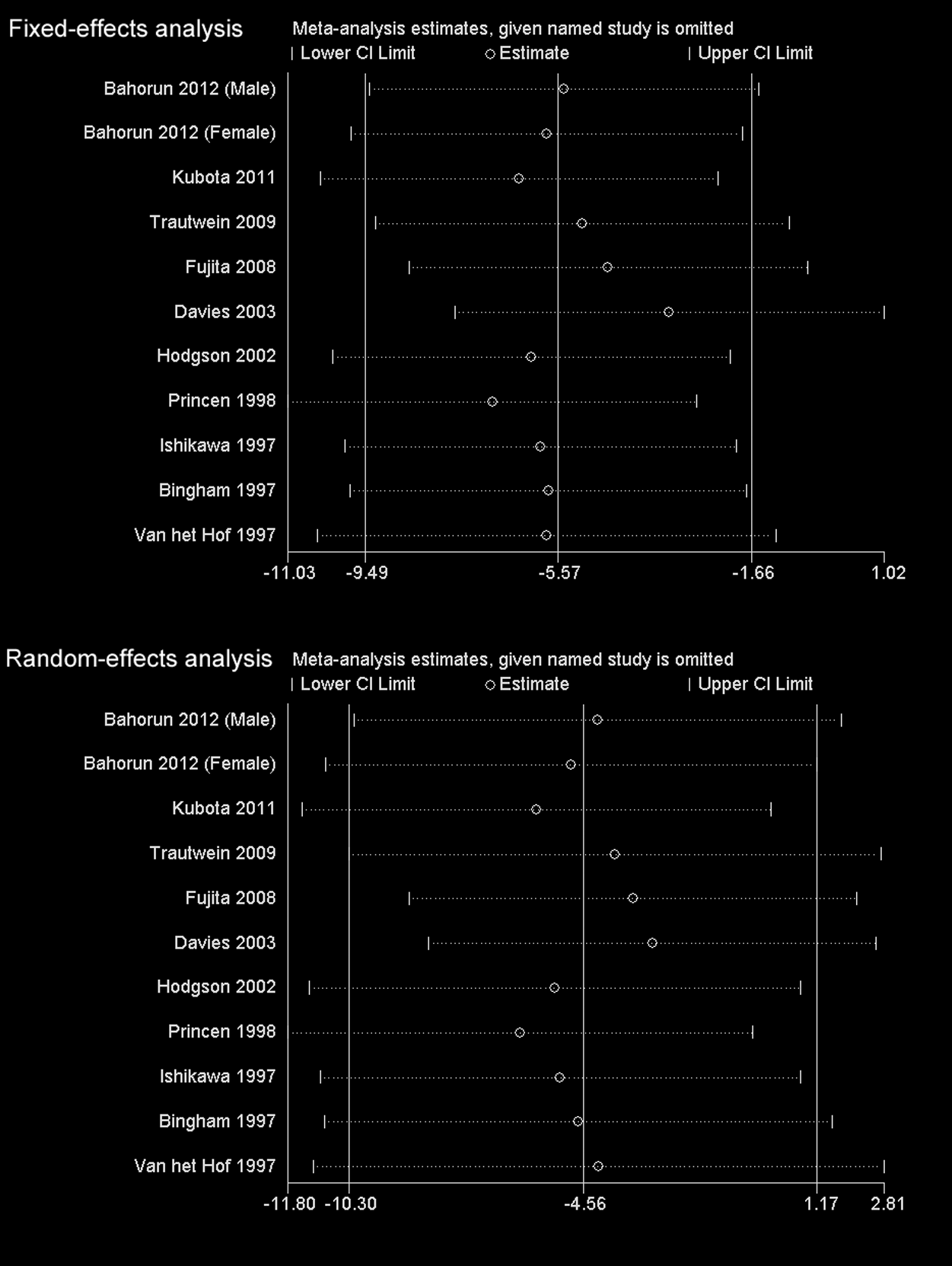

Supplement: Figure S9 — Sensitivity analyses of effect of black tea on LDL-C concentrations in healthy subjects. A meta-analysis was done with STATA software (Version 11; StataCorp, College Station, TX). Weight of each study was shown by sizes of data markers in the analysis. The diamond represents the overall estimated outcome and the results were calculated using a fixed-effects or random-effects model. WMD, weighted mean difference. (TIF) [file pone.0107711.s009.tif]
