# Supplementary material for: Effect of Black Tea Consumption on Blood Cholesterol: A Meta-Analysis of 15 Randomized Controlled Trials
Source: PLoS One. 2014 Sep 19;9(9):e107711. doi: 10.1371/journal.pone.0107711 (PMC4169558; doi:10.1371/journal.pone.0107711)
Supplement: Appendix S1 — A separate search strategy designed for each electronic database. (DOC) [file pone.0107711.s010.doc]

1. PubMed (<http://www.ncbi.nlm.nih.gov/pubmed/>, through to July 2014):

black tea[Title/Abstract] OR black tea extract[Title/Abstract] OR catechin[Title/Abstract] OR catechins[Title/Abstract] OR EGCG[Title/Abstract] OR camellia sinensis[Title/Abstract] OR theaflavin[Title/Abstract] OR tea polyphenols[Title/Abstract] AND (Randomized Controlled Trial[ptyp] AND "humans"[MeSH Terms]).

2. Embase and MEDLINE (<http://www.embase.com/>, through to July 2014):

‘black tea’:ab,ti OR ‘black tea extract’:ab,ti OR catechin:ab,ti OR catechins:ab,ti OR EGCG:ab,ti OR ‘camellia sinensis’:ab,ti OR theaflavin:ab,ti OR ‘tea polyphenols’:ab,ti AND [randomized controlled trial]/lim AND [humans]/lim.

3. Cochrane (<http://www.thecochranelibrary.com/view/0/index.html>, through to July 2014):

(black tea:ab,ti or black tea extract:ab,ti or catechin:ab,ti or catechins:ab,ti or EGCG:ab,ti or camellia sinensis:ab,ti or theaflavin:ab,ti or tea polyphenols:ab,ti) and randomized controlled trials, combined with the options of “trials”.
