# Supplementary material for: Effect of Black Tea Consumption on Blood Cholesterol: A Meta-Analysis of 15 Randomized Controlled Trials
Source: PLoS One. 2014 Sep 19;9(9):e107711. doi: 10.1371/journal.pone.0107711 (PMC4169558; doi:10.1371/journal.pone.0107711)
Supplement: Appendix S2 — Supporting PRISMA Flow Diagram. (PDF) [file pone.0107711.s011.pdf]

PRISMA 2009 Flow Diagram

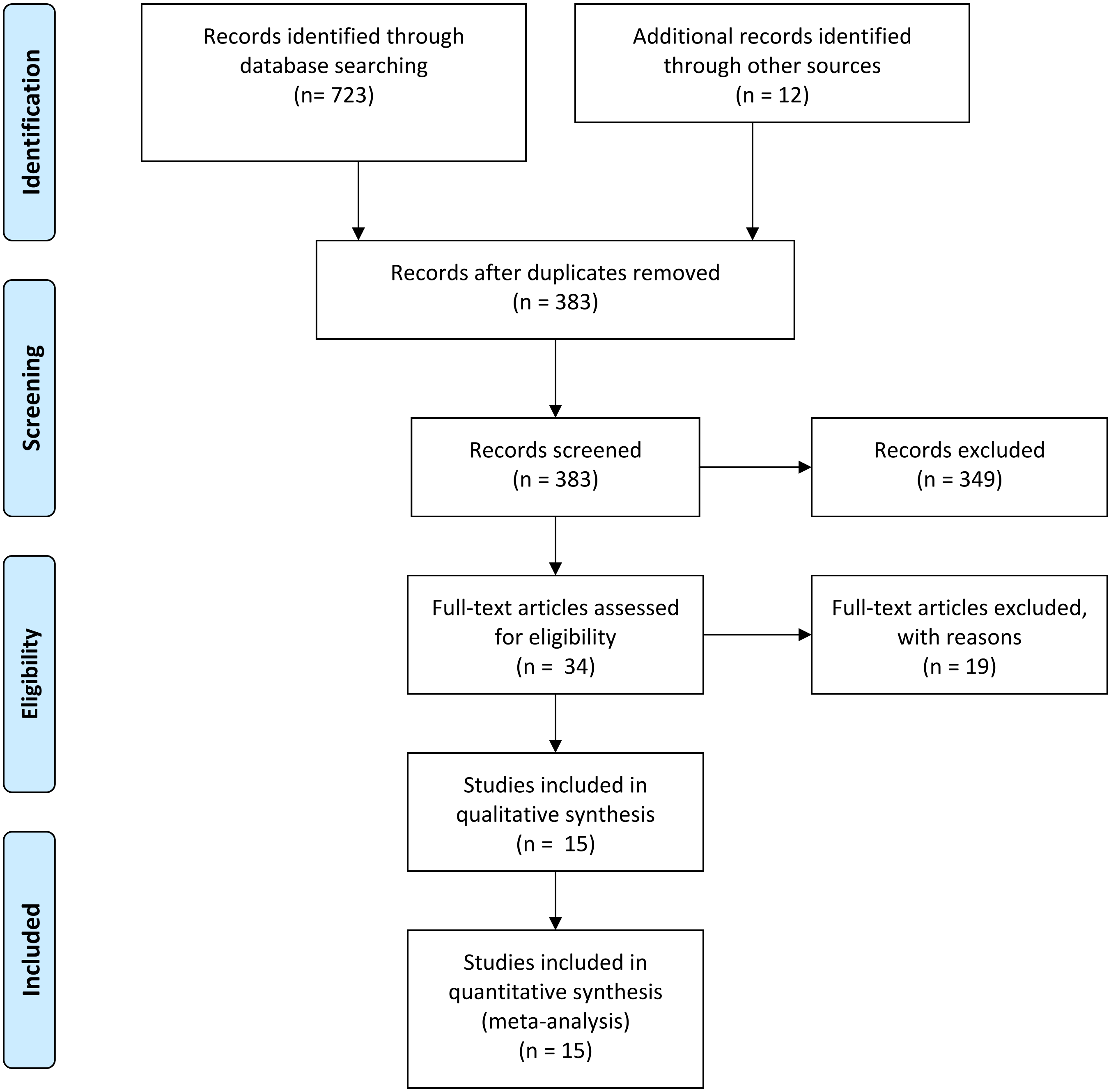

From: Moher D, Liberati A, Tetzlaff J, Altman DG, The PRISMA Group (2009). *P*referred *R*eporting *I*tems for *S*ystematic Reviews and *M*eta-*A*nalyses: The PRISMA Statement. PLoS Med 6(6): e1000097. doi:10.1371/journal.pmed1000097

For more information, visit www.prisma-statement.org.
